# Management of Massive Rivaroxaban Overdose With Acetaminophen and Isosorbide Mononitrate Overdose

**DOI:** 10.7759/cureus.34019

**Published:** 2023-01-20

**Authors:** Bradley Casey, Abigail Daniels, Amol Bahekar, Divyang Patel, Alejandro Chapa-Rodriguez

**Affiliations:** 1 Internal Medicine, Cape Fear Valley Medical Center, Fayetteville, USA; 2 Emergency Medicine, Campbell University School of Osteopathic Medicine, Lillington, USA; 3 Cardiology, Cape Fear Valley Medical Center, Fayetteville, USA; 4 Critical Care Medicine, Cape Fear Valley Medical Center, Fayetteville, USA

**Keywords:** hemorrhage, isosorbide mononitrate, acetaminophen toxicity, rivaroxaban, drug overdose

## Abstract

Direct oral anticoagulants (DOACs) have been used more frequently for the prevention and management of thromboembolic disease in comparison to their predecessors. DOACs provide greater ease of administration, shorter half-lives, less monitoring, and fewer drug-drug interactions. With the rise of DOACs such as rivaroxaban, the opportunity for abuse also increases. Therefore, standardization of care based on rivaroxaban misuse must also be explored, an area in which there is not ample information. We present a case where a patient consumed a stockpile of her home medications in hopes to commit suicide. A 64-year-old female presented to the emergency department due to the ingestion of rivaroxaban 5,000 mg along with ingestion of acetaminophen 30,000 mg and isosorbide mononitrate 1000 mg in the setting of intentional self-harm with multiple declarations of being classified as Do Not Resuscitate. There have been documented cases of rivaroxaban overdose, however, there are no documented cases with levels of ingestion reaching 5,000 mg along with signs of severe bleeding. Our case study reviews the previously documented management of rivaroxaban abuse and the treatment that was given to our patient in the setting of extreme anticoagulant ingestion.

## Introduction

Rivaroxaban, also known as Xarelto, is a factor Xa inhibitor used in the management of non-valvular atrial fibrillation, deep vein thrombosis, and pulmonary embolism [1]. Its metabolism is performed by two main metabolic enzymes, the major enzyme of metabolism being CYP3A and the minor enzyme of metabolism being CYP2J [2]. Rivaroxaban’s half-life is five to nine hours, with an increased half-life of 11 to 13 hours in elderly persons and a peak concentration in two to four hours [3]. Isosorbide mononitrate, also known as Imdur, is a medication used for the management and treatment of chronic angina [4]. Isosorbide mononitrate's metabolism is performed by the metabolic enzyme CYP3A4, and the half-life is five to six hours with an onset of action of 30-45 minutes and a peak serum accumulation of 30-60 minutes [4]. The main adverse effects associated with the use of isosorbide mononitrate are the development of hypotension and bradycardia, even in small doses [4].

Throughout the literature, there are a few case reports associated with rivaroxaban overdose and the management strategies performed. In the case studies that have been published, the maximum known dosage of rivaroxaban has not been more than the dose that was ingested by our patient. Furthermore, although there are documented cases of severe bleeding with the ingestion of rivaroxaban, the amount ingested is not known, and therefore management is unclear. Here, we present a case of a severe Xarelto overdose that had significant bleeding.

## Case presentation

A 64-year-old 93 kg female with a past medical history including hypertension, history of multiple pulmonary embolisms, paroxysmal non-valvular atrial fibrillation, heart failure with preserved ejection fraction, coronary artery disease (CAD) status post percutaneous coronary intervention and drug-eluting stent (DES) most recently for angioplasty to the left circumflex artery, cerebral vascular accident (CVA) with residual left-sided weakness, a major depressive disorder with post-traumatic stress disorder (PTSD), and previous suicide attempts, presents to the emergency department (ED) for intentional self-harm and a suicide attempt. The patient had been discharged the previous day with home health due to the unavailability of an open bed for inpatient rehabilitation. Upon discharge, the patients’ medications were filled for a 90-day supply, including rivaroxaban and isosorbide mononitrate. The following day, the home health nurse arrived for their assessment. The home health nurse reported that the patient had multiple old prescription bottles beside her, including rivaroxaban, isosorbide mononitrate, and an over-the-counter acetaminophen bottle.

Upon arrival in the ED, it was found the patient had ingested rivaroxaban 5000 mg, acetaminophen 30,000 mg, as well as isosorbide mononitrate 1000 mg. The patient's review of systems was positive for nausea, lightheadedness, headache, and suicidal ideation. A physical exam revealed oozing epistaxis of the bilateral nares, bilateral lower extremity non-pitting edema, a right forearm oozing hematoma, and chronic residual left-side weakness. The patients’ initial vital signs were within normal limits. The patient’s labs of significance included a white blood count of 15.7 × 10*3/µL (normal 4.5-12.5 × 10*3/µL), hemoglobin of 11 g/dl (normal 12.0-16.0 g/dL), hematocrit of 34.7% (normal 36.0-48.0 %), PT of 38.5 seconds (normal 9.7-12.4 seconds), INR of 3.9 (normal 0.9-1.1), PTT of 77.3 seconds (normal 24.3-34.6 seconds), salicylate level of less than 2.8 mg/dl (normal 2.8-20.0 mg/dL), and acetaminophen level of 263.4 µg/ml (normal 10.0-30.0 µg/mL). The patient’s ethanol and urine drug screens were both negative. A head computed tomography (CT) scan and chest X-ray (CXR) revealed no acute pathology.

The patient arrived at the ED within 30 minutes of ingestion and was given activated charcoal. Activated charcoal-sorbitol 50 g/240 mL suspension for a total of 100 g was given due to the ingestion of toxic amounts of medication. Due to the excessive amount of Tylenol ingestion, N-acetylcysteine 150 mg/kg bolus followed by a 15 mg/kg/hour infusion was given for acetaminophen toxicity. Four-factor human prothrombin complex concentrate (Kcentra) 4770 units was given, as well as Phytonadione (Vitamin K) 10 mg. While still in the ED, the patient was coughing, which resulted in hemoptysis that was controlled with nebulized tranexamic acid (TXA). The management of this patient was complicated by continuous bleeding for approximately four hours after the patient’s initial presentation and after the perfusion of Kcentra. The decision to begin an Andexanet alfa bolus of 13,950 mg (150 mg/kg) was made and followed by a 15 mg/kg infusion. The patient was then admitted to the intensive care unit for monitoring.

Throughout treatment and during the patient’s hospital stay, repeat laboratory data 24 hours post-treatment showed an acetaminophen level of less than 2 µg/ml. PT and INR eventually returned to baseline levels around 36 hours after the administration of reversal agents. In Table 1, all of the reversal agents were given between 10:59 p.m. and 3:37 a.m. Hemoglobin remained stable throughout hospitalization and never dropped below 10 g/dl. The patient’s bleeding stopped approximately 10 hours after the administration of Kcentra, and by 11:45 a.m. (Table 1), the bleeding had stopped. The patient was eventually transferred from the ICU to the psychiatry floor and eventually discharged home on rivaroxaban and isosorbide mononitrate.

**Table 1:** Shows the trend of labs from the patient at the time of emergency department arrival (10:59 PM row 1) until transferred out of the intensive care unit (10:50 AM row 8). All of the reversal agents were given between 10:59 PM row 1 and 3:37 AM row 2.

	Protime (normal 9.7–12.4 seconds)	International normalized ratio (INR) (Normal 0.9–1.1)	Activated partial thromboplastin time (aPTT) (normal 24.3–34.6 seconds)	Hemoglobin (normal 12.0–16.0 g/dl)
10:59 PM	38.5 (H)	3.9 (H)	77.3 (H)	11.0 (L)
3:37 AM	25.6 (H)	2.5 (H)		10.9 (L)
11:45 AM	18.9 (H)	1.8 (H)	33	10.8 (L)
5:05 PM	17.5 (H)	1.7 (H)	28.8	11.0 (L)
5:05 PM	17.7 (H)	1.7 (H)		11.0 (L)
1:16 AM	14.3 (H)	1.3 (H)		10.8 (L)
5:30 AM	13.0 (H)	1.2 (H)		
10:50 AM	12.3	1.1		11.0 (L)

## Discussion

The first orally dosed direct factor Xa inhibitor was rivaroxaban [5]. It binds directly and reversibly to Factor Xa and competitively inhibits factor Xa, demonstrating more than 10,000-fold selectivity for factor Xa than other related serine proteases [5]. About one-third of the medication is eliminated by the kidneys, with the rest by the liver, and after approximately 15 hours, the expected anticoagulant effect has decreased [6,7]. There is only a minimal effect seen from rivaroxaban after 24 hours [7]. In the case of acute rivaroxaban overdose, the major concern is bleeding, and supportive measures were the standard of care for the majority of patients prior to May 2018 [8]. In May of 2018, Andexanet alfa was approved as an antidote for rivaroxaban [2]. Andexanet alfa is a modified human factor Xa decoy protein with a half-life of approximately one hour [2]. It is difficult to monitor rivaroxaban overdose by laboratory value, as the gold standard is measured by liquid chromatography-tandem mass spectrometry [9]. Liquid chromatography was not present at our facility to accurately measure the levels of Rivaroxaban. PT and aPTT are affected without any linearity, depending on the reagents used, but normal PT and aPTT seem to exclude relevant rivaroxaban plasma levels [10-12]. Charcoal should be administered if the patient presents between 30 and 60 minutes after ingestion [13]. Since a specific antidote is not available, the administration of prothrombin complex concentrate seems to be the best option in cases of severe bleeding [7,14]. Due to high plasma protein binding, hemodialysis has no role in the management of the bleeding associated with the use of rivaroxaban [15].

Isosorbide is a nitrate that exerts its pharmacologic effect by releasing nitric oxide (NO), an endothelium-derived relaxing factor (EDRF) [16]. NO is endogenously produced in the endothelium to dilate the blood vessels [16]. At therapeutic levels, it predominately dilates the venous capacitance vessels but also the coronary arteries and the arterioles [17]. The elimination half-life of isosorbide mononitrate is five to six hours [18]. The symptoms of isosorbide overdose may arise from its vasodilation property, causing profound systemic hypotension, heart block with bradycardia, and syncope [18]. Due to insufficient human research on isosorbide mononitrate toxicity management is conservative.

Acetaminophen toxicity is the second most common cause of liver transplantation worldwide and the most common cause of liver transplantation in the US [19]. Acetaminophen is rapidly absorbed from the gastrointestinal tract and reaches therapeutic levels in 30 minutes to 2 hours [19]. Overdose levels peak at four hours, and acetaminophen has an elimination half-life of two hours [19]. The treatment of an acetaminophen overdose depends on when the drug was ingested. If the patient presents within one hour of ingestion, GI decontamination may be attempted with activated charcoal [19]. All patients with high levels of acetaminophen need admission and treatment with N-acetyl-cysteine (NAC); NAC is fully protective against liver toxicity if given within eight hours after ingestion [19]. If our patient had arrived after 60 minutes, she would not have received activated charcoal. Since two-thirds of rivaroxaban is metabolized by the liver, we may have seen a longer-lasting anticoagulant effect if acetaminophen caused significant hepatic damage.

Three cases of massive rivaroxaban overdose were discovered in the literature review: 1400 mg, 1800 mg, and 1960 mg [20-22]. None of the cases reviewed had an intake near the 5000 mg of rivaroxaban that our patient consumed. At our facility, we did not have access to liquid chromatography-tandem mass spectrometry, but we were able to trend PT and INR. Due to the patient receiving charcoal in the ED shortly after ingestion, she did not require transplant consultation from the acetaminophen overdose. The patient's bleeding stopped shortly after the administration of reversal agents, so she did not require any packed red blood cells.

## Conclusions

This case report allows us to explore the treatment and management of an unprecedented presentation of rivaroxaban, isosorbide mononitrate, and acetaminophen overdose resulting in major hemorrhage. Although treatment and management strategies for acetaminophen overdose are well described, the same cannot be said for the treatment and management of an isosorbide overdose and massive rivaroxaban overdose. The strategies used in the case were based upon limited evidence, and the decisions made throughout this case will hopefully allow for a standardization of care in the setting of any level of anticoagulant use or misuse.
